# Comparative evaluation of ChatGPT and gemini responses to patient-oriented questions on breast cancer

**DOI:** 10.1371/journal.pone.0358325

**Published:** 2026-09-16

**Authors:** Yeliz Yilmaz Bozok, Nihan Acar, Murat Kemal Atahan, Cem Karaali

**Affiliations:** Department of General Surgery, İzmir Atatürk Training and Research Hospital, İzmir Katip Çelebi University, İzmir, Turkey; University of Pisa, ITALY

## Abstract

**Background:**

Breast cancer, the most common malignancy among women, remains a major global public health concern. With the rapid growth of artificial intelligence–based language models, it is essential to evaluate their potential roles in patient education. This study compared ChatGPT and Gemini in responding to patient-oriented questions about breast cancer and its surgical treatment regarding scientific accuracy, clarity, and unnecessary detail.

**Methods:**

Forty frequently asked questions were collected from Turkish online sources. Both models were queried under identical conditions on August 13, 2025, and the responses were anonymized for blinded evaluation. Four general surgeons experienced in breast surgery independently assessed each response using a five-point Likert scale across three domains: scientific accuracy, clarity, and unnecessary detail.

**Results:**

Response lengths were recorded and compared. ChatGPT achieved significantly higher median scores than Gemini in scientific accuracy [4.75 (3.50–5.00) vs. 4.25 (3.50–5.00); p < 0.001], clarity [4.75 (3.50–5.00) vs. 4.25 (3.25–5.00); p = 0.005], and unnecessary detail [5.00 (5.00–5.00) vs. 4.50 (3.50–5.00); p < 0.001]. The overall median score was 4.83 (4.08–5.00) for ChatGPT and 4.33 (3.50–4.92) for Gemini (p < 0.001). Gemini’s responses were significantly longer (244 ± 84 vs. 170 ± 47 words; p < 0.001).

**Conclusion:**

Both models demonstrated acceptable overall performance. However, ChatGPT’s higher scores and shorter, more focused responses indicate that it may be a more efficient tool for addressing breast cancer–related patient questions. In their current form, these models should not be used for diagnostic or clinical decision-making purposes. AI-generated health information should be used only under expert supervision and for educational or supportive purposes.

## Introduction

Breast cancer, the most common malignancy among women, remains a major public health concern in both developed and developing countries [1]. Despite significant technological advances in screening and treatment, it continues to be one of the leading causes of cancer-related mortality among women worldwide [2].

Globally, there is a marked inequality in the burden of breast cancer. Although the incidence is higher in high-income countries, the mortality rate is lower due to the effectiveness of early detection programs and better access to treatment. In contrast, low- and middle-income countries experience increasing mortality rates, largely attributable to diagnostic delays and limited healthcare resources [3].

Delays in the diagnosis and treatment of breast cancer have been associated with poor quality of life and reduced survival outcomes [4,5]. A multicenter study conducted in Türkiye focusing on the causes of diagnostic and treatment delays in breast cancer identified key patient-related factors, including lack of knowledge (12.4%), fear of losing the breast (8.9%), and fear of death (9.8%). These findings highlight that patient-related factors play a predominant role in diagnostic delays [6].

Access to accurate medical information is crucial for reducing patient anxiety and guiding individuals toward timely medical consultation. In this context, large language models (LLMs) have recently emerged as modern tools that can potentially enhance health literacy and support patient education. Health literacy plays a critical role in the effectiveness of patient education materials. Previous recommendations from the National Institutes of Health, the US Department of Health and Human Services, and the American Medical Association suggest that patient-directed educational materials should ideally be written at approximately a sixth-grade reading level to improve accessibility and comprehension [7].

Previous reports suggest that a substantial proportion of internet users seek medical information online, with rates approaching 80% in some studies [8,9]. In addition, ChatGPT was estimated to have nearly 300 million weekly active users and approximately 3.8 billion monthly visits as of November 2024 [10]. AI-generated medical information may increasingly influence patient understanding, treatment expectations, and healthcare-related decision-making processes [11,12].

However, their rapid proliferation has raised important questions regarding the accuracy and reliability of the information they provide. Growing concerns exist that AI-based systems may generate or disseminate misleading health information at a large scale [13].

In recent years, bibliometric and Altmetric analyses have increasingly been used to evaluate the scientific visibility and online dissemination of medical information. These approaches provide complementary insights into the relationship between academic publications, social media engagement, and public interest in health-related topics [14].

The aim of this study was to comparatively evaluate the responses generated by ChatGPT and Gemini to patient-oriented questions on breast cancer and its surgical management in terms of scientific accuracy, clarity, and the level of unnecessary detail.

## Methods

This cross-sectional comparative study evaluated patient-oriented questions identified through Google and YouTube searches in the local language and by reviewing the “frequently asked questions” sections of health institutions. When compiling the question list related to breast cancer and its surgery, the following categories were considered: symptoms and diagnosis, risk factors and prevention, imaging methods, disease course and prognosis, treatment options, and treatment-related side effects. After merging and simplifying similar questions, a final list consisting of 40 questions was obtained.

In this study, two LLMs, ChatGPT (GPT 5, OpenAI, USA) and Gemini 2.5 Flash (Google DeepMind, United Kingdom), were used. To standardize the evaluation conditions, separate user accounts were created for each LLM, and all responses were obtained within the same date and time range (August 13, 2025, 17:00–20:00). In addition, searches and model interactions were performed using incognito mode to minimize potential personalization effects related to previous browsing activity or search history. The questions were directed to each model without any additional prompts or role instructions, and each question was asked in a new session. This approach prevented any possible contextual influence from preceding or subsequent questions. The responses were transferred to anonymized booklets by the study designer without any modification, ensuring blinded evaluation by the raters. The second booklet was delivered to the raters at least seven days after the first one was completed to minimize potential recall bias. In addition, the word count of each response generated by the LLMs was recorded for every question.

Four general surgery specialists experienced in breast cancer and breast surgery, including two professors and two associate professors, participated as raters. The AI-generated responses were assessed across three main dimensions: scientific accuracy, clarity, and level of unnecessary detail. A five-point Likert-type scale (1–5) was used for each dimension. The scale items were clearly defined to describe the meaning of each level in detail. For example, a score of “1” represented responses containing major scientific errors, unclear or confusing language, or excessive irrelevant information; whereas a score of “5” indicated responses fully consistent with current guidelines, clear, coherent, and limited to essential information. These detailed definitions were included in the evaluation form to improve consistency among raters.

As this research focused solely on the analysis of AI-generated content and did not involve any patient data, medical records, archived samples, or identifiable personal information, ethics committee approval and informed consent were not required.

### Statistical analysis

The distribution characteristics of the data were evaluated using the Shapiro–Wilk test. Since the score-related variables did not show normal distribution, paired comparisons were performed using the non-parametric Wilcoxon signed-rank test. These variables were expressed as median (minimum–maximum) values. When a significant difference was found and the median values were equal, the direction of the difference was interpreted based on the comparison of mean ranks. The Wilcoxon signed-rank test was used for paired groups; effect size was reported using Cohen's d coefficient. Median differences between paired measurements and their 95% confidence intervals were estimated using the Hodges–Lehmann method. The response length data showed normal distribution; therefore, comparisons were performed using the paired-samples t-test. The effect size was reported using Cohen's d coefficient. All analyses were performed using IBM SPSS Statistics version 24.0 (IBM Corp., Armonk, NY, USA). A p value of <0.05 was considered statistically significant.

### Results

**Fig 1:**
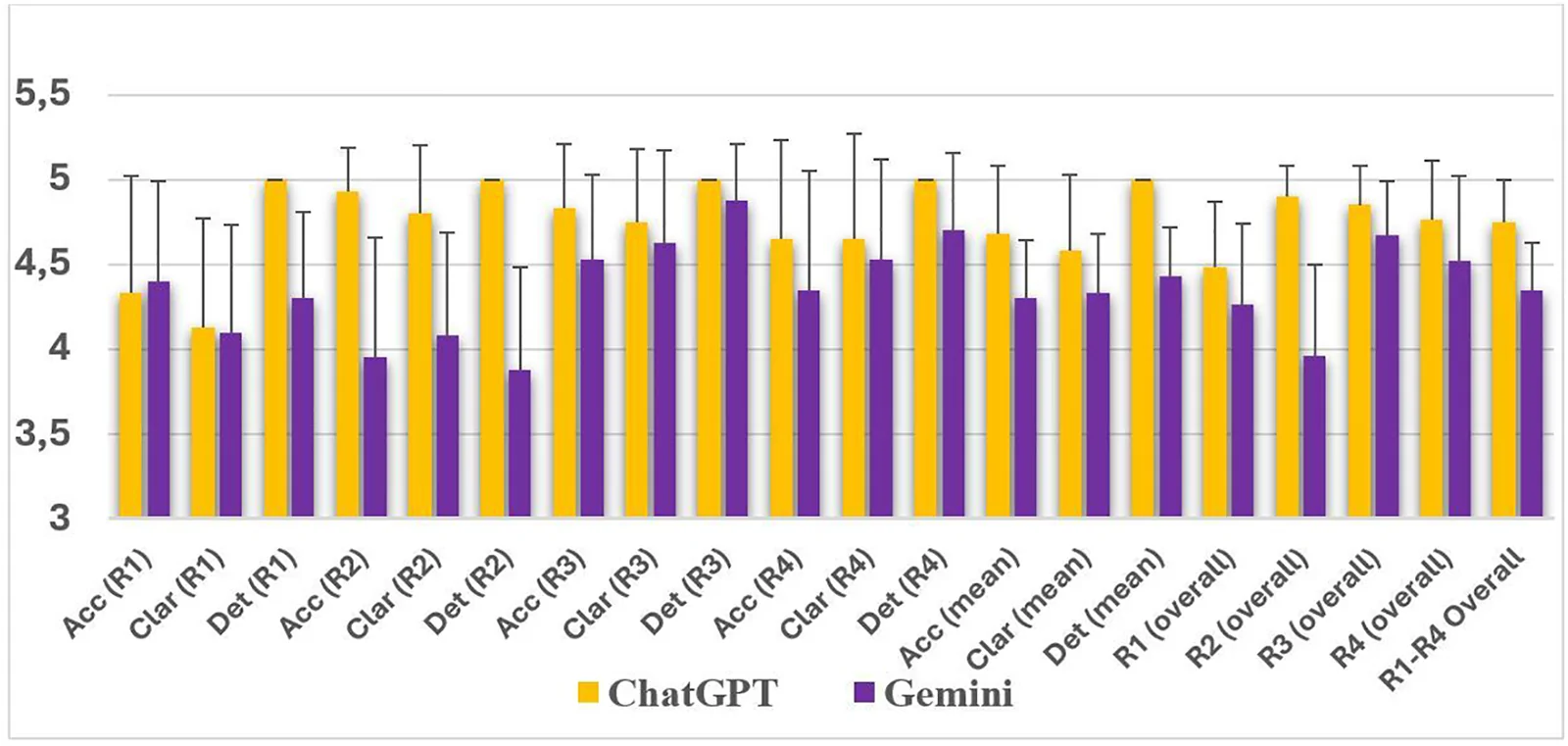
Comparison of ChatGPT and Gemini across three evaluation domains: scientific accuracy (Acc), clarity (Clar), and unnecessary detail (Det), as rated by four evaluators (R1–R4). Bars show median scores with error bars for variability.

Based on the aggregated scores of four raters, the median score of ChatGPT for scientific accuracy was significantly higher than that of Gemini [4.75 (3.50–5.00) vs. 4.25 (3.50–5.00), p < 0.001]. In the clarity dimension, the median score of ChatGPT [4.75 (3.50–5.00)] was also higher than that of Gemini [4.25 (3.25–5.00), p = 0.005]. Similarly, in the level of unnecessary detail, ChatGPT showed a significantly higher median score [5.00 (5.00–5.00) vs. 4.50 (3.50–5.00), p < 0.001]. Comparisons of scientific accuracy, clarity, and level of unnecessary detail are shown in Fig 1.

When overall mean scores were analyzed, the median score of ChatGPT was 4.83 (4.08–5.00), while that of Gemini was 4.33 (3.50–4.92), and the difference was statistically significant (p < 0.001). Detailed comparisons for each evaluation domain and rater are presented in Table 1. Gemini generated significantly longer responses than ChatGPT (244 ± 84 vs. 170 ± 47 words, p < 0.001). Detailed comparisons of response lengths are presented in Table 2.

**Table 1:** Comparison of ChatGPT and Gemini across three evaluation domains: scientific accuracy, clarity, and level of unnecessary detail, for each evaluator (R1–R4) and overall results.

	ChatGPT	Gemini	Test statistic	p value
Scientific accuracy (R1)	4 (3–5)	4 (3–5)	Z = −0.645	0.519
Clarity (R1)	4 (3–5)	4 (3–5)	Z = −0.209	0.835
Level of unnecessary detail (R1)	5 (5–5)	4 (3–5)	Z = −5.112	<0.001
Scientific accuracy (R2)	5 (4–5)	4 (3–5)	Z = −4.950	<0.001
Clarity (R2)	5 (4–5)	4 (3–5)	Z = −4.498	<0.001
Level of unnecessary detail (R2)	5 (5–5)	4 (3–5)	Z = −5.417	<0.001
Scientific accuracy (R3)	5 (4–5)	5 (4–5)	Z = −3.000	0.003
Clarity (R3)	5 (4–5)	5 (3–5)	Z = −1.147	0.251
Level of unnecessary detail (R3)	5 (5–5)	5 (4–5)	Z = −2.236	0.025
Scientific accuracy (R4)	5 (3–5)	4 (3–5)	Z = −2.149	0.032
Clarity (R4)	5 (3–5)	5 (3–5)	Z = −0.887	0.375
Level of unnecessary detail (R4)	5 (5–5)	5 (4–5)	Z = −3.464	<0.001
Scientific accuracy (mean)	4.75 (3.5-5)	4.25 (3.5-5)	Z = −4.056	<0.001
Clarity (mean)	4.75 (3.5-5)	4.25 (3.25-5)	Z = −2.831	0.005
Level of unnecessary detail (mean)	5 (5–5)	4.5 (3.5-5)	Z = −5.467	<0.001
R1 (overall)	4.33 (3.67-5)	4 (3.33-5)	Z = −2.453	0.014
R2 (overall)	5 (4.33-5)	4 (3–5)	Z = −5.286	<0.001
R3 (overall)	5 (4.33-5)	4.66 (3.67-5)	Z = −2.543	0.011
R4 (overall)	5 (3.67-5)	4.66 (3.33-5)	Z = −2.660	0.008
Overall	4.83(4.08-5)	4.33 (3.5-4.92)	Z = −4.878	<0.001

**Table 2:** Word count comparison of ChatGPT and Gemini responses for 40 breast cancer questions.

	Question	ChatGPT	Gemini
1	What are the symptoms of breast cancer?	**198**	**164**
2	Is breast pain a sign of cancer?	**103**	**246**
3	Does performing a biopsy on a breast lump cause cancer to spread?	**124**	**185**
4	Is every palpable lump in the breast cancer?	**114**	**121**
5	Can breast inflammation or abscess turn into cancer?	**144**	**285**
6	Can men get breast cancer?	**138**	**514**
7	Can young people get breast cancer?	**155**	**287**
8	Can breast cancer occur during pregnancy?	**172**	**283**
9	Can a woman who has had breast cancer become pregnant?	**189**	**273**
10	Who are the individuals at high risk for breast cancer?	**197**	**303**
11	Should I have a genetic test if there is a family history of breast cancer?	**200**	**267**
12	Does having a BRCA mutation increase the risk of breast cancer?	**131**	**232**
13	Does smoking cause breast cancer?	**148**	**206**
14	Does eating too many sweets increase the risk of breast cancer?	**143**	**179**
15	Does hormone use increase the risk of breast cancer?	**173**	**186**
16	Do underarm deodorants cause breast cancer?	**174**	**269**
17	Is the radiation exposure from mammography harmful for breast cancer?	**129**	**202**
18	Is MRI better than mammography for breast imaging?	**265**	**136**
19	Does breast cancer metastasize or spread to other organs?	**102**	**139**
20	Where does breast cancer most commonly spread?	**117**	**107**
21	Is breast cancer contagious?	**70**	**169**
22	Can breast cancer be completely cured?	**151**	**309**
23	Can breast cancer recur after treatment?	**177**	**284**
24	Which type of breast cancer is the most lethal?	**155**	**194**
25	What is the prognosis of triple-negative breast cancer?	**246**	**289**
26	How does HER2-positive breast cancer progress?	**236**	**372**
27	What is the average survival of breast cancer patients?	**254**	**209**
28	What is the average survival time in stage IV breast cancer?	**156**	**136**
29	Do I need to follow a special diet for breast cancer?	**250**	**378**
30	Are there any medications to prevent breast cancer?	**184**	**209**
31	Do herbal or alternative remedies help in breast cancer treatment?	**116**	**272**
32	Is chemotherapy mandatory for breast cancer treatment?	**198**	**316**
33	Is it better to receive chemotherapy before surgery?	**215**	**241**
34	Can breast cancer be cured with chemotherapy alone without surgery?	**150**	**186**
35	Does chemotherapy for breast cancer carry a risk of death?	**216**	**277**
36	Does chemotherapy for breast cancer cause hair loss?	**160**	**218**
37	Is there any way to prevent hair loss during breast cancer treatment?	**203**	**314**
38	Is immunotherapy used in breast cancer treatment?	**129**	**135**
39	What are the side effects or risks of radiotherapy in breast cancer?	**253**	**372**
40	Can breast implants be placed during breast cancer surgery?	**172**	**314**
	Mean ± SD	170.18 ± 47.49	244.45 ± 83.88
	p-value (Paired-samples t-test)	<0.001	
	Test stat	−5.897	

For Rater 1, no statistically significant difference was observed between ChatGPT and Gemini in scientific accuracy (p = 0.519) or clarity (p = 0.835). However, ChatGPT scored significantly higher in the level of unnecessary detail [median 5.00 (5.00–5.00) vs. 4.00 (3.00–5.00), p < 0.001]. The overall median scores of Rater 1 were 4.33 (3.67–5.00) for ChatGPT and 4.00 (3.33–5.00) for Gemini, with a statistically significant difference (p = 0.014).

For Rater 2, ChatGPT achieved higher median scores than Gemini across all dimensions. In scientific accuracy [5.00 (4.00–5.00) vs. 4.00 (3.00–5.00)], clarity [5.00 (4.00–5.00) vs. 4.00 (3.00–5.00)], and level of unnecessary detail [5.00 (5.00–5.00) vs. 4.00 (3.00–5.00)] the differences were statistically significant (p < 0.001, p < 0.001, p = 0.003 respectively). The overall median scores of Rater 2 were 5.00 (4.33–5.00) for ChatGPT and 4.00 (3.00–5.00) for Gemini (p < 0.001).

For Rater 3, a significant difference favoring ChatGPT was found in scientific accuracy (p = 0.003). Although the medians were equal [5.00 (4.00–5.00) vs. 5.00 (4.00–5.00)], the difference was supported by mean rank analysis. No significant difference was found in clarity [5.00 (4.00–5.00) vs. 5.00 (3.00–5.00); p = 0.251]. In the level of unnecessary detail, as in scientific accuracy, equal medians [5.00 (5.00–5.00) vs. 5.00 (4.00–5.00)] but higher mean ranks indicated a difference favoring ChatGPT (p = 0.025). The overall median scores of Rater 3 were 5.00 (4.33–5.00) for ChatGPT and 4.66 (3.67–5.00) for Gemini (p = 0.011).

For Rater 4, ChatGPT demonstrated higher performance than Gemini in scientific accuracy [5.00 (3.00–5.00) vs. 4.00 (3.00–5.00); p = 0.032]. No statistically significant difference was observed in clarity, as the median values were equal for both models [5.00 (3.00–5.00) vs. 5.00 (3.00–5.00); p = 0.375]. In terms of the level of unnecessary detail, although the median scores were equal [5.00 (5.00–5.00) vs. 5.00 (4.00–5.00)], mean rank analysis indicated a statistically significant difference favoring ChatGPT (p = 0.000532). Overall, the median score was higher for ChatGPT than for Gemini [5.00 (3.67–5.00) vs. 4.66 (3.33–5.00); p = 0.008].

## Discussion

This study comprehensively evaluated artificial intelligence-generated responses to patient-oriented questions regarding breast cancer and its surgical management. Both models demonstrated overall high performance; however, ChatGPT outperformed Gemini significantly in terms of scientific accuracy, clarity, level of unnecessary detail, and overall mean score.

As in many other fields, artificial intelligence applications are rapidly expanding within healthcare [15]. AI assistance now extends from diagnostic and imaging processes to reducing clinicians’ workloads and enhancing patient engagement. Despite these advantages and the trust these systems often inspire, the possibility of errors and the uncontrolled spread of misinformation remain among the most critical concerns in medical contexts.

Tan et al. evaluated ChatGPT’s responses to patient questions about glaucoma and emphasized that, despite promising results, the potential for generating incorrect or misleading information may pose risks for patients [16]. Similarly, Yalamanchili et al. found that while large language models could serve as an effective alternative to online health resources for radiation oncology patients, their readability remained suboptimal [17]. Similarly, a recent comparative study evaluating ChatGPT, Gemini, and Perplexity reported concerns regarding the reliability of AI-generated medical information and emphasized that these systems may still produce factually inaccurate or difficult-to-read responses [18]. In another study focusing on lung cancer and surgical management, ChatGPT achieved scores above 4.5 across all dimensions of scientific adequacy, clarity, and accuracy [19]. However, the authors cautioned that the model’s reliance on outdated training data and the potential for “hallucinations” (fabricated or incorrect information) may result in misinformation. In contrast, our study identified only minor phrasing differences or trivial factual inaccuracies, with no clinically significant misinformation detected. Nevertheless, the possibility of such errors, particularly in the context of medical decision-making, should not be underestimated given their potential clinical implications.

Another key finding was that Gemini produced significantly longer responses compared to ChatGPT, which may represent a potential disadvantage. For instance, for the question “Can men get breast cancer?”, ChatGPT generated a 138-word response, whereas Gemini produced 514 words. While Gemini’s overall performance remained acceptable, the longer and less focused responses may have contributed to its lower scores. Similar findings have been reported in previous Turkish-language comparative studies using the same LLMs in different medical fields, where Gemini consistently generated longer responses than GPT-based models [20,21]. Tong et al. reported opposite findings in a different domain, noting that Gemini generated shorter responses than ChatGPT [22]. These inconsistencies suggest that response length variations depend on factors such as model version, subject domain, and evaluation conditions. The observed performance differences may also be related to differences in the models’ development processes, technological maturity, and response-generation characteristics. However, given that this comparison was conducted within a specific language, clinical domain, and expert evaluator group, the observed superiority should not be assumed to persist under different evaluation conditions. To improve readability and prevent information overload, integrating response-length control mechanisms, such as word or character limits, into user interfaces could help optimize the outputs of these systems.

Our study contributes to the limited literature evaluating AI-generated responses to patient questions about breast cancer and its surgical treatment. Piao et al. conducted a similar study in China comparing ChatGPT, ERNIE Bot, and ChatGLM and found comparable overall performances among the models, although ChatGPT was rated higher for human-like empathy [23]. The authors emphasized that such tools are suitable for addressing general informational queries but remain unreliable for complex clinical topics. Likewise, Roldan-Vasquez et al. found ChatGPT’s responses to breast surgery–related questions to be accurate and reliable, with only minor, clinically insignificant errors [24].

To the best of our knowledge, this study represents one of the first comparative evaluations of large language model responses to patient-oriented questions regarding breast cancer and its surgical management in the Turkish language. In addition, the blinded evaluation performed by multiple specialists under standardized assessment conditions represents a methodological strength of the study.

This study has several limitations. First, patient questions were collected exclusively in the local language (Turkish), which may limit cross-linguistic comparability. Previous studies evaluating Turkish internet-based patient education materials have reported important limitations regarding the readability, quality, and reliability of online health information [25]. Therefore, the findings of the present Turkish-language study may not be directly generalizable to other languages and healthcare settings. Future studies conducted in multiple languages with similar designs could enhance the generalizability of findings. Second, large language models are continuously updated and may produce variable outputs over time, potentially affecting response consistency. Therefore, the findings of this study reflect the performance of the model versions available at the time of data collection and may not fully represent their current or future performance. Nevertheless, because both models were evaluated during the same time period using identical questions and assessment methods, the present findings provide a standardized comparative assessment of their performance at that specific point in time. In addition, only two widely used LLMs were evaluated in this study. The inclusion of additional AI models in future studies may provide a broader comparison of AI-generated patient education content. Lastly, this evaluation relied solely on expert assessments without incorporating patient perspectives. Integrating patient feedback into future evaluations would provide a more holistic understanding of the real-world educational impact of these models.

## Conclusion

This research represents one of the first comparative evaluations of large language model–generated responses to patient-oriented questions on breast cancer and its surgical management. Both models achieved high scores overall, but ChatGPT demonstrated significantly better performance than Gemini in scientific accuracy, clarity, and focus. Beyond the importance of early diagnosis, patient understanding and confidence at the outset of treatment play a crucial role in therapeutic success. In this context, both models may serve as useful supplementary tools for patient education and awareness. However, considering its advantage in response length and content focus, ChatGPT may represent a more efficient option. Nevertheless, in their current form, the outputs of both models should not be used as diagnostic instruments or clinical decision-making tools. Given the inherent risk of misinformation, all AI-generated content must be interpreted under expert supervision and utilized solely for supportive and educational purposes. Future studies incorporating multilingual designs and patient participation will be essential to better define the reliability and clinical utility of AI-based language models in healthcare communication.

## Supporting information

S1 File Supplementary Tables 1–8. (PDF)
