# Supplementary material for: Comparative evaluation of ChatGPT and gemini responses to patient-oriented questions on breast cancer
Source: PLoS One. 2026 Sep 16;21(9):e0358325. doi: 10.1371/journal.pone.0358325 (PMC13580998; doi:10.1371/journal.pone.0358325)
Supplement: Supplementary file 1 [file pone.0358325.s001.pdf]

Supplementary Table 1. Rater 1 scores for ChatGPT responses

No:	Scientific accuracy	Clarity	Level of unnecessary detail
1	3	3	5
2	4	3	5
3	5	4	5
4	4	4	5
5	5	4	5
6	5	5	5
7	4	5	5
8	4	4	5
9	5	5	5
10	5	5	5
11	5	5	5
12	4	4	5
13	5	5	5
14	5	5	5
15	4	4	5
16	3	3	5
17	5	4	5
18	4	4	5
19	5	5	5
20	4	4	5
21	5	5	5
22	5	4	5
23	4	4	5
24	4	4	5
25	4	4	5
26	4	4	5
27	5	4	5
28	3	4	5
29	5	5	5
30	5	3	5
31	5	5	5
32	4	4	5
33	3	3	5
34	4	4	5
35	3	4	5
36	4	4	5
37	5	4	5
38	4	3	5
39	5	4	5
40	4	4	5

Supplementary Table 2. Rater 1 scores for Gemini responses

No:	Scientific accuracy	Clarity	Level of unnecessary detail
1	5	4	4
2	4	3	4
3	5	3	4
4	5	5	4
5	4	4	4
6	5	4	4
7	4	4	4
8	3	4	4
9	4	4	4
10	4	4	5
11	5	5	5
12	4	4	4
13	5	5	5
14	5	5	5
15	3	4	4
16	4	4	4
17	4	4	4
18	5	5	5
19	5	4	5
20	5	4	4
21	5	4	5
22	5	4	5
23	4	5	4
24	4	3	4
25	4	3	4
26	4	3	4
27	5	5	5
28	5	5	5
29	4	4	4
30	4	4	4
31	5	4	5
32	4	4	4
33	4	3	3
34	4	4	4
35	4	4	4
36	5	5	5
37	5	5	5
38	4	4	4
39	5	4	4
40	4	4	4

Supplementary Table 3. Rater 2 scores for ChatGPT responses

No:	Scientific accuracy	Clarity	Level of unnecessary detail
1	4	5	5
2	5	5	5
3	5	5	5
4	5	5	5
5	5	4	5
6	5	5	5
7	5	5	5
8	5	5	5
9	5	5	5
10	5	5	5
11	5	4	5
12	5	5	5
13	5	5	5
14	5	5	5
15	5	5	5
16	5	4	5
17	5	5	5
18	5	5	5
19	5	5	5
20	5	5	5
21	5	5	5
22	5	5	5
23	5	5	5
24	5	5	5
25	5	5	5
26	5	5	5
27	5	5	5
28	5	5	5
29	5	5	5
30	5	4	5
31	5	5	5
32	5	5	5
33	4	4	5
34	5	5	5
35	5	5	5
36	5	5	5
37	5	5	5
38	4	4	5
39	5	4	5
40	5	4	5

Supplementary Table 4. Rater 2 scores for Gemini responses

No:	Scientific accuracy	Clarity	Level of unnecessary detail
1	5	5	5
2	4	4	4
3	4	4	4
4	3	4	4
5	4	4	4
6	4	4	3
7	4	4	4
8	4	4	4
9	4	4	4
10	5	5	5
11	3	4	4
12	3	4	4
13	4	4	3
14	4	4	4
15	4	4	4
16	4	5	3
17	5	5	4
18	3	3	3
19	3	4	4
20	4	4	4
21	5	5	3
22	3	3	3
23	4	4	3
24	4	3	3
25	4	4	4
26	5	5	5
27	5	5	5
28	5	4	4
29	3	4	4
30	4	4	4
31	5	4	4
32	3	3	3
33	3	3	3
34	4	4	4
35	3	4	4
36	4	5	4
37	4	4	4
38	3	3	4
39	5	5	5
40	4	4	4

Supplementary Table 5. Rater 3 scores for ChatGPT responses

No:	Scientific accuracy	Clarity	Level of unnecessary detail
1	4	4	5
2	5	4	5
3	5	5	5
4	5	5	5
5	5	4	5
6	5	5	5
7	4	5	5
8	5	5	5
9	5	5	5
10	5	5	5
11	5	4	5
12	5	5	5
13	5	5	5
14	5	5	5
15	5	5	5
16	4	4	5
17	5	5	5
18	5	5	5
19	5	5	5
20	5	5	5
21	5	5	5
22	5	5	5
23	5	5	5
24	5	5	5
25	5	5	5
26	5	5	5
27	5	5	5
28	4	5	5
29	5	5	5
30	5	4	5
31	5	5	5
32	5	5	5
33	4	4	5
34	5	5	5
35	5	5	5
36	5	5	5
37	5	5	5
38	4	4	5
39	5	4	5
40	4	4	5

Supplementary Table 6. Rater 3 scores for Gemini responses

No:	Scientific accuracy	Clarity	Level of unnecessary detail
1	4	5	5
2	5	5	5
3	5	4	5
4	5	5	5
5	4	4	5
6	4	4	5
7	5	5	5
8	5	5	5
9	5	5	5
10	5	4	5
11	5	5	5
12	4	5	5
13	4	4	5
14	4	5	4
15	4	5	5
16	4	4	5
17	5	5	5
18	5	5	5
19	4	5	5
20	4	4	5
21	5	5	5
22	5	5	5
23	5	5	5
24	4	5	5
25	4	5	5
26	4	3	4
27	4	5	5
28	4	4	5
29	4	5	5
30	5	4	5
31	5	4	5
32	5	4	4
33	4	4	4
34	5	4	4
35	5	5	5
36	5	5	5
37	5	5	5
38	4	5	5
39	4	5	5
40	5	5	5

Supplementary Table 7. Rater 4 scores for ChatGPT responses

No:	Scientific accuracy	Clarity	Level of unnecessary detail
1	3	4	5
2	5	4	5
3	5	5	5
4	5	5	5
5	5	4	5
6	5	5	5
7	4	5	5
8	5	5	5
9	5	5	5
10	5	5	5
11	5	4	5
12	4	5	5
13	5	5	5
14	5	5	5
15	5	4	5
16	4	3	5
17	5	5	5
18	5	5	5
19	5	5	5
20	4	5	5
21	5	5	5
22	5	5	5
23	5	5	5
24	4	4	5
25	5	5	5
26	5	5	5
27	5	5	5
28	4	5	5
29	5	5	5
30	4	3	5
31	5	5	5
32	5	5	5
33	4	4	5
34	5	5	5
35	4	5	5
36	5	5	5
37	5	5	5
38	3	3	5
39	5	5	5
40	4	4	5

Supplementary Table 8. Rater 4 scores for Gemini responses

No:	Scientific accuracy	Clarity	Level of unnecessary detail
1	4	5	5
2	5	5	5
3	5	5	5
4	4	4	5
5	5	5	5
6	5	5	5
7	5	4	4
8	5	4	5
9	5	4	5
10	4	5	5
11	3	4	4
12	5	5	5
13	4	4	4
14	5	5	5
15	4	5	5
16	4	5	4
17	4	5	5
18	3	4	4
19	5	5	5
20	5	5	5
21	5	4	5
22	3	4	4
23	4	4	5
24	4	4	5
25	4	5	5
26	5	5	5
27	5	5	5
28	5	5	5
29	5	5	5
30	5	5	5
31	5	5	5
32	3	3	4
33	4	3	4
34	4	4	4
35	4	4	5
36	4	5	4
37	4	5	5
38	3	4	4
39	5	5	5
40	4	4	4
